# Feasibility of a novel neurofeedback system: a parallel randomized single-blinded pilot study

**DOI:** 10.1038/s41598-023-44545-1

**Published:** 2023-10-13

**Authors:** Dávid Horváth, János Négyesi, Melinda Rácz, Tamás Győri, Zsolt Matics, Artyom Puskin, János Csipor, Levente Rácz

**Affiliations:** 1 Department of Kinesiology, Hungarian University of Sports Science, Budapest, Hungary; 2 Fit4Race Kft., Budapest, Hungary; 3 Neurocognitive Research Center, National Institute of Mental Health, Neurology and Neurosurgery, Budapest, Hungary; 4 Department of Medicine and Science in Sports and Exercise, Tohoku University Graduate School of Medicine, Sendai, Japan; 5 Research Centre for Natural Sciences, Eötvös Loránd Research Network, Budapest, Hungary; 6 MindRove Kft., Győr, Hungary; 7 János Szentágothai Doctoral School of Neurosciences, Semmelweis University, Budapest, Hungary; 8 Selye János Doctoral College for Advanced Studies, Semmelweis University, Budapest, Hungary; 9 Department of Psychology and Sport Psychology, Hungarian University of Sports Science, Budapest, Hungary; 10 Faculty of Electrical Engineering and Informatics, Budapest University of Technology and Economics, Budapest, Hungary

**Keywords:** Biomedical engineering, Neurophysiology

## Abstract

Neurocognitive assessment tools have been proposed to optimize, maintain, and improve perceptual-cognitive performance. Here, we investigated the feasibility and efficacy of a novel neurofeedback system, neuroMoon (nM), on cognitive abilities compared with one of the most popular perceptual-cognitive training (PCT) tools both in sports and rehabilitation called NeuroTracker (NT). Thirty-one young athletes performed a comprehensive battery of cognitive tests from the Vienna Test System before and after a 12-session computer-based cognitive training program using nM (n = 11, age 22.6 ± 3.8 years), nM sham (CON, n = 10, age 20.3 ± 1.2 years) or NT (n = 10, age 20.5 ± 1.7 years) device. A series of repeated-measures ANOVA was performed to detect changes in cognitive abilities in response to the training. Participants had faster median reaction time in both the color-naming and word-reading conditions of the Stroop test (all p < 0.005), regardless of group. Regarding the task switching test, statistical analysis indicated faster working time and mean reaction time of the incongruent stimuli, repetition task, and shifting task (all p < 0.005), nevertheless, these changes were also regardless of group. In addition, we found fewer omitted (pre: 17.5 ± 8.3, post: 6.4 ± 1.5, d = 1.311) and more correct (pre: 261.6 ± 36.1, post: 278.6 ± 38.7, d = − 1.020) post-intervention answers in the determination test, regardless of group. Finally, participants in each group performed the digit span backward test with larger post (6.42 ± 1.54) vs. pre (5.55 ± 1.43) scores following the PCT (d = − 0.801). Overall, PCT with nM as compared with NT induced similar results in cognitive abilities suggesting its potential to be used to achieve and maintain better mental performance. However, considering that the sham stimulation also induced similar improvements in cognitive abilities, future studies should clearly determine the cognitive measures that could benefit from NF training.

## Introduction

Achieving the best sports performance requires not only strength, endurance, and sport-specific training but also the improvement of perceptual and decision-making skills. Implementing these types of exercises into the training regime could help athletes to process the most important information at the right time to make accurate decisions during the competitions. Indeed, a previous meta-analysis^1^ revealed that experts are better than nonexperts in perceptual-cognitive skills suggesting the need for their improvement in elite sports. Consequently, in the last two decades, there has been substantial interest in identifying the effects of perceptual-cognitive skills in expert sports performance (for reviews, see^2–5^). For example, a previous study examined how different perceptual-cognitive skills interact during performance and found that skilled players made more anticipation and decision-making in situational probabilities and pattern recognition when compared with their less-skilled counterparts^6^.

Neurocognitive assessment tools have been proposed to optimize, maintain, and improve perceptual-cognitive performance. NeuroTracker (NT) (promoted and sold by Faubert Applied Research Centre, University of Montréal, and CogniSens Athletics Inc.) is one of the most popular perceptual-cognitive training (PCT) tools both in sports and rehabilitation. A previous systematic review^7^ reported that some attentional skills like working memory, sustained attention, processing speed or inhibition might improve with NT training, however, its scientific proof for real-world perceptual-cognitive skills is premature due to the many methodological concerns in published studies. Nevertheless, NT is widely used by professional clubs in the NFL, NBA, and NHL, what is more, the U.S. military has been also reported to implement NT into their cognitive practice.

Neurofeedback (NF) is a technique that utilizes real-time displays of brain activity, most commonly measured via electroencephalography (EEG), to promote self-regulation of brain function. This closed-loop method helps individuals to control or modify their cortical activity through learned self-regulation, with the aim of improving alertness, reducing anxiety and enhancing cognitive abilities such as attention, memory, and behavior. There is a growing body of literature that supports the effectiveness of NF, particularly in the field of cognitive functions and attention-deficit/hyperactivity disorder (ADHD)^8, 9^. Among the various NF protocols, sensorimotor (SM) NF training appears to be one of the most effective^10^ since SMR is the dominant frequency of the integrated thalamocortical somatosensory and somatomotor pathways and its operant conditioning may result in improved control over the system^11^, modulating attention^12^. Theta (T) rhythm (4–7 Hz) has been linked to neurological and psychological functions in the limbic system, including the control of arousal, affective and mental states^13^, and also to working memory^12^. NF protocols that target the stimulation of SMR and the depression of T have been successfully applied prior to our work. In a study, SMR/T NF training improved the mental performance of elderly people with mild cognitive impairment^14^. Improvement of cued recall performance and focused attentional processing accuracy have been reported, as well^12^. As it was also shown, the stimulation of SMR without a concurrent rise in T activity reduced the number of commission errors and had a positive effect on perceptual sensitivity and P300 event-related potential amplitude^15^, and led to the reduction of omission errors and reaction time variability^16^. Another paper^13^ reports the beneficial effect of this latter method on microsurgical skills. The suppression of T has been found effective in enhancing the performance of dancers in relation to an alpha/T NF paradigm^17^. Regarding performance training in sports, the potential of this technique has been demonstrated in relation to mental work speed and efficiency as well as self-reported engagement^18^, to the artistry and execution quality of balance beam performance along with the self-reported increase of energy and self-awareness in gymnasts^19^, moreover, to the reduction of anxiety in swimmers^20^ and arousal in archers^21^, respectively. Given the promising results of NF in cognitive improvement, we hypothesized that this technique might also be useful in other athletes to enhance their cognitive performance.

The majority of NF studies use regular EEG caps for data acquisition but the number of papers featuring portable headsets is increasing. The most frequently used devices are the Emotiv EPOC^22–25^, InteraXon Muse^26–28^, and NeuroSky MindWave^29, 30^ headsets. Common properties of these devices are the limited number of working electrodes (EPOC: 14, Muse: 5, MindWave: 1) and that these electrodes do not require the use of additional conductive substances (besides saline, at most) for proper functioning. An additional challenge of lightweight EEG hardware is the lack of working electrodes above the areas that are most frequently used in SMR/T NF procedures, most notably, Cz/Pz locations. We wanted to know whether it is possible to implement such a system based on brain signals recorded above the frontal and occipital cortices. The role of theta-beta ratio (TBR) (i.e., the ratio of power within the 4–7 Hz and 13–30 Hz ranges) of signals measured above the frontal areas have been reported in relation to attentional control^31–33^, emotion regulation^34, 35^, grit (perseverance)^36^ and correct time perception^37^ negatively and to mind wandering positively^38–40^. Despite of not being primary sites of motor planning or execution, occipital areas have also been mentioned in the literature in the context of motor control and SMR NF. In a motor adaptation learning task, one of the best predictors of learning was the beta power measured above occipital/parieto-occipital areas^41^. In another research study^42^, resting-state SMR power in parieto-occipital areas predicted the success of NF training. Moreover, occipital T brain waves have also been reported in relation to an NF procedure targeting the mitigation of generalized anxiety^43^.

In the present parallel randomized single-blinded pilot study, we investigated the efficacy of a novel NF system, called neuroMoon (nM) on cognitive abilities by examining the differences between the improvements after nM vs. NT training. We hypothesized that the level of cognitive improvements after nM vs. NT training will not differ. To address this hypothesis, participants performed the Digit Span Backwards (DSB) cognitive test and a comprehensive battery of cognitive tests from the Vienna Test System (VTS) before and after a 12-session computer-based cognitive training program using nM or NT device. Our study fits under the current efforts about developing techniques that could support achieving and maintaining better mental performance under challenging conditions in intricate environments.

## Methods

### Participants

Sample size calculations (G*Power 3.1.7^44^) revealed that a minimum sample size of 24 participants would be appropriate to detect significant differences between the experimental and control groups, assuming a moderate effect size, type I error of 0.05, and a power of 0.80. In this parallel randomized single-blinded pilot study, 31 participants with no reported neurological deficits or SM impairment were randomly assigned to either nM (n = 11, age 22.6 ± 3.8 years, 2 female), nM sham (CON, n = 10, age 20.3 ± 1.2 years, 1 female) or NT (n = 10, age 20.5 ± 1.7 years, 1 female) group. Participation was free of charge; the participants did not receive honoraria for their participation. The participants received both verbal and written explanations of the experimental protocol that was in accordance with the declaration of Helsinki. After this, participants signed the informed consent document. All experimental protocols were approved by the University (Hungarian University of Sports Science, Budapest, Hungary) Ethical Committee (Approval No. TE-KEB/No36/2022).

### Study design

**Figure 1:**
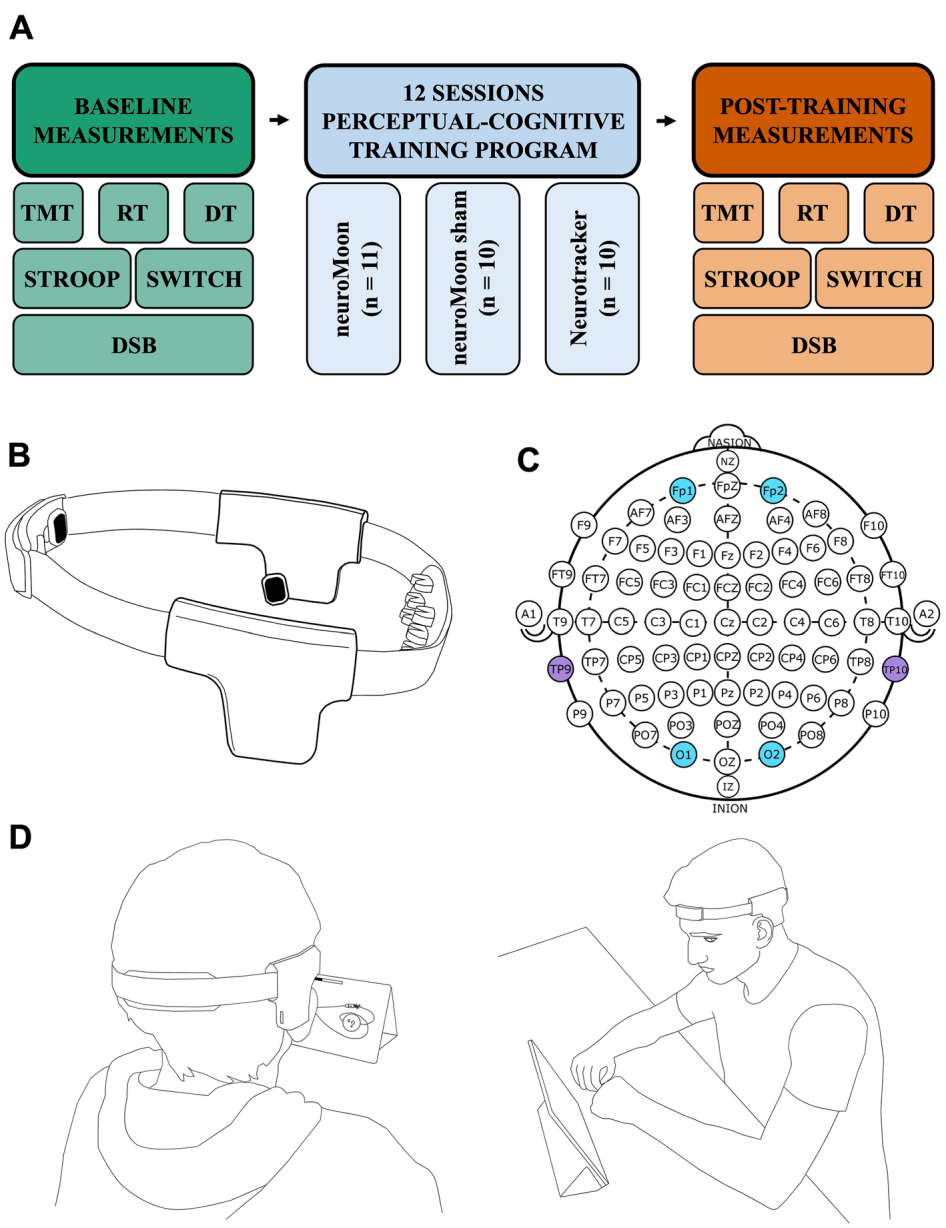
Schematic illustration of the neuroMoon (nM) neurofeedback system. (**A**) Schematic illustration of the experimental design. Prior to the start of the training program, each participant had to complete a test consisting of five tasks. During the 12-sessions training program, the participants were split into three groups, one using nM, nM sham and NeuroTracker (respectively). After the completion of the training, participants were asked to repeat the test. (**B**) Picture of the nM EEG headset (alpha version). (**C**) The position of the 4 EEG electrodes of the nM device was designed according to the International 10–20 system for EEG electrode placement. Working electrodes and references are highlighted in blue and purple, respectively. (**D**) Experimental setup in a typical setting. *TMT* trail-making test, *STROOP* Stroop test, *RT* reaction test, *SWITCH* task-switching test, *DT* determination test, *DSB* digit span backwards test. Shapes and images were created by JN, MR, and JC using Microsoft Office Power Point 2021 software (Version: 16.74, Microsoft, Redmond, Washington, USA) and Inkscape 1.1.2 (b8e25be833, 2022-02-05); https://inkscape.org/), respectively.

Figure 1A provides a schematic illustration of the experimental design. Participants had individual experimental procedures at our research facility (Fit4Race, Budapest, Hungary), which is specialized to test and train motorsport athletes. Participants were asked not to drink alcohol 24 h before and during the testing session and not to drink coffee in the mornings of the testing sessions. The tests consisted of the Digit Span Backwards cognitive test and 5 cognitive tasks from the VTS^45^. To signal auditory cues, a headset was provided during the experiment.

The following week, participants began the 4-week computer-based cognitive training program three times a week with 48–72 h of rest in between each session. Each participant in each group was asked not to perform any cognitive-skills development training during the research study. We also asked them not to change their daily routine or eating habits. The post-test was performed 2 days after the last training session and was identical to the baseline measurements.

### neuroMoon system

The nM device, developed by MindRove, is an EEG-based NF headset prototype (Fig. 1B). To ensure the reliability and accuracy of the device, MindRove used the same printed circuit board panel as it is used in their commercially available EEG devices^46^. The device utilizes a high-end EEG chip. This chip is a 24-bit, 8-channel analog-to-digital converter (ADC) that is specifically designed for use in EEG and other bio-potential measurement applications. The sampling rate of the device is 250 SPS, the gain is 12. It has a high input impedance and low noise, which ensures high-quality data. The chip is also known for its high common-mode rejection ratio and low input bias current, which helps to reduce noise and interference in the EEG signals. The device is powered by a rechargeable LiPo battery that lasts for 4–6 h.

The nM device is designed to be flexible and adjustable to the participant's head size. The device consists of four rigid components that are integrated into a headband, which includes the frontal, occipital, reference and DRL electrodes, the electronics, and the battery. The first component, located in the front of the headband, contains electrodes that contact with the forehead of the wearer, providing EEG measurements from the Fp1 and Fp2 locations, according to the 10–20 system (Fig. 1C). The 10–20 system provides a standardized method for the placement of electrodes on the scalp, ensuring that EEG signals are obtained from consistent and well-defined locations. The second component, located in the back of the device, contains electrodes that are specifically configured to penetrate through the hair and contact with the skin, providing EEG measurements from O1 and O2 locations. The third component of the device houses the electronics and reference electrode (TP10), while the fourth component houses the battery and DRL electrode (TP9).

The position of the 4 EEG electrodes of the nM headset was designed according to the International 10–20 system for EEG electrode placement. In order to obtain accurate and reliable EEG signals, it is important to minimize the contact impedance between the electrodes and the skin. To achieve this, electrodes feature conductive fabrics sewn with platinum-iridium wire as the contact layers. The use of conductive fabrics as the contact layer increases the surface area of contact between the electrode and the skin, which in turn reduces the contact impedance. Platinum-iridium wire, on the other hand, has a high electrical conductivity, which also helps to reduce contact impedance. This electrode design maximizes the signal-to-noise ratio and provides a good-quality EEG signal which is essential for NF training. The electrodes are dry electrodes, which do not require any gel or paste to be applied to the skin. Dry electrodes are known for their ease of use and convenience, as they can be quickly and easily applied to the skin without the need for additional preparation.

### Testing procedures

Cognitive abilities were identified using the Digit Span Backwards test and the VTS, which is a widely-used objective measure of various psychological constructs that appeared to have the potential to provide information on the effects of certain factors on athlete cognitive performance (for review see^47^) even for car racing drivers^48^. Here, we selected and used 5 tests from a comprehensive cognitive test package of VTS to determine the effects of a 12-session computer-based cognitive training program using NT, nM, or nM sham (CON) on participants’ cognitive performance. Each test was preceded by a familiarization. The VTS system provided feedback to the participants in case of incorrect answers and did not allow them to proceed to the testing session until the correct answer was given.

#### Trail-making test (TMT)

The trail-making test is a widely-used, easily accessible neuropsychological instrument that provides the examiner with information on a wide range of cognitive skills such as visuomotor processing speed and cognitive flexibility. Its background, administration, guidelines, and interpretations are well described in a ~ 2-decades-old protocol^49^. Briefly, in part A, the numbers 1 to 25 appeared on the screen in a random arrangement. The participant was asked to click on them in sequential order as quickly as possible. Part B uses the numbers 1 to 13. The participant was required to click the numbers and letters alternately in ascending order as quickly as possible. The test took about 5 min and was preceded by a familiarization trial.

#### Stroop test (STROOP)

The Stroop test was used to examine the participants’ cognitive flexibility (or switching ability). The detailed explanation of the test is described by the distributor^45^ and also in our previously published manuscript^48^. Briefly, two conditions were used without interfering influences (congruent stimuli) to determine baseline performance (BL) and were related to the two interference (IF) conditions i.e., (a) ”color naming interference” and (b) ”word reading interference” (incongruent stimuli). Participants had to press the appropriate button on the test panel as quickly as possible. The test took ~ 10 min and was preceded by 10–10 familiarization trials for each condition. The reading and naming interference, the reaction time, and the % of incorrect answers during the interference conditions were analyzed.

#### Reaction test (RT)

The reaction test measures reaction time and motor time in response to simple and complex visual (light) or acoustic (sound) signals. The test contained simple light signals (yellow or red light), a tone, or a combination of the two stimuli (yellow and tone or yellow and red). During the test, the participant was required to press and release a button as quickly as possible only when a yellow light signal was presented simultaneously with a tone. The test took about 5–10 min. The mean reaction time was calculated and used for further analysis.

#### Task-switching test (SWITCH)

The VTS’s task-switching test measures flexible task-switching ability as an aspect of the executive functions. During the task, a sequence of circles and triangles appeared on the monitor in either light or dark grey. The participant was asked to react to either the shape (circle or triangle) or brightness (light or dark) of the figure by pressing the appropriate button of the VTS panel. The test took about 12 min. The task switching speed and accuracy were used in the statistical analysis.

#### Determination test (DT)

The detailed explanation of the determination test is described elsewhere^45, 48^. Briefly, it is used to evaluate participants’ reactive stress tolerance, attention, and reaction speed in situations requiring continuous, swift, and varying responses to rapidly changing visual, and acoustic stimuli. After a familiarization session, participants were presented with color stimuli and acoustic signals and were asked to react by pressing the appropriate buttons on the response panel. The participants needed to sustain continuous, rapid, and varying responses to rapidly changing stimuli. The test is adaptive, which means that the software continuously adapted the level of challenge based on the performance of each participant. The test took about 6 min. The number of correct, incorrect, and omitted answers was evaluated and then was used in the statistical analysis.

#### Digit span backwards (DSB)

The digit span backwards test is a test that measures complex working memory^50^. During the test, the examiner read out a series of single-digit numbers, leaving a one-second pause between the numbers. The task of the participant was to repeat the spoken sequence of numbers in reverse order of the way they heard it before. Each section has 4 attempts, at least two of which must be answered correctly by the participant in order to move on to the next section. Omissions or substitutions when reciting the sequence of numbers are considered errors. The final span index is the length at which the participant was able to repeat correctly two out of four attempts.

### Training procedures

#### neuroMoon

The NF protocol used is aimed at increasing the sensorimotor rhythm-theta ratio (SMR/T). The SMR band (or low beta components^16^ due to its more frequent use, we use the former term throughout this article) falls between 12 and 15 Hz and the T band in the 4–7 Hz range, respectively. When this ratio increases, the user receives positive feedback which encourages them to continue with the training. EEG signals for SMR/T training were recorded from the FP1–2 and the O1–2 channels according to the International 10–20 system. The protocol is aimed at increasing the SMR/T ratio, meaning that SMR was stimulated while T waves were suppressed. A high pass filter with a cutoff frequency of 0.5 Hz and a notch filter with a center frequency of 50 Hz were applied to eliminate low-frequency noise and signal electrical interference, respectively. The sampling rate was 250 Hz.

During the processing of EEG signals, an important step is the batching and analysis of the incoming signals. Each batch is composed of a four-second interval, during which 500 EEG samples are collected. To ensure continuity and smooth transitions, we applied a 100-sample overlap between consecutive batches. This four-second duration allows for a granular examination of the signals and the subsequent calculation of their power across the desired frequency bands, a critical aspect of our game development application.

For the segmentation and subsequent analysis, the code employs the Welch method, a widely recognized technique for estimating the power spectral density (PSD) of time series data like EEG signals. The Welch method divides the EEG signal into overlapping segments using a specified windowing function, in this case, the Hann window, which mitigates spectral leakage. For each segmented epoch, a periodogram is computed. This is achieved by first applying the chosen window (Hann) to the segment, followed by computing its Fast Fourier Transform (FFT). The FFT operation plays a pivotal role as it translates the EEG signal from the time domain into the frequency domain. This transformation illuminates the distribution of the signal's power across various frequencies. The obtained periodograms from all segments are then averaged, producing a consistent estimate of the PSD.

Having estimated the PSD, our focus then shifts to power calculation across desired frequency bands. This is where the Simpson method comes into play. The Simpson method is an integral component of our approach. Given the PSD estimates, it calculates the total power within the desired frequency range. This is done by integrating the PSD values using Simpson's rule, which offers a more accurate approximation compared to standard summation techniques.

Taken together, the Welch method for PSD estimation and the Simpson integration for power calculation offer a thorough insight into the EEG signals. The clear distinction of power across specific frequency bands aids in fine-tuning our game development application, allowing for real-time adaptability based on the user's EEG data.

The NF training method takes the form of a video game that runs on an Android tablet (Fig. 1D). The game features a spaceship that orbits two planets on a fixed path. The spaceship has a default speed that may be accelerated by increasing the SMR/T ratio compared to the baseline, thus providing NF to the user. The acceleration of the spaceship is also shown by a progress bar on the screen. The session started with a calibration phase for 30 s that was followed by 3 consecutive NF trials, each lasting for 210 s separated by 1-min resting phases. Baseline measurements were taken to establish the neural activity of each participant, which were used as a reference point for later NF sessions. These measurements typically included the recording of the EEG signals while the participant was in a relaxed, resting state. It was ensured that the participant sat comfortably and did not experience any discomfort during the calibration session.

The velocity of the spaceship was calculated using a formula that takes the default speed of the spaceship, the new SMR/T ratio value and the calibrated value obtained during the calibration phase into account:

$$v={v}_{default}\left(1+2\frac{x-{x}_{cal}}{{x}_{cal}}\right)$$

wherein ${x}_{cal}$ is the average density value of a power spectrum of SMR/T ratio calculated during the calibration period; $x$ is the density of a power spectrum of SMR/T ratio calculated during the NF at a specific time interval; $v$ is the actual speed of the spaceship; and ${v}_{default}$ is the default speed of the spaceship.

#### neuroMoon sham

The CON protocol was performed to exclude non-specific effects of NF training and was almost identical to the nM protocol except for the calculation of the velocity of the spaceship that was based on random values instead of the SMR/T ratio and the calibration value. During the training sessions, the velocity is assigned a random value from three possible ranges in a periodic fashion:

$$v\in \left\{\begin{array}{c}\left[\begin{array}{ccc}0.1& \dots & 0.2\end{array}\right], k \mathrm{mod} 11=0, 1, \dots 5 \\ \left[\begin{array}{ccc}0.2& \dots & 0.5\end{array}\right], k \mathrm{mod} 11=6, 7, 8\\ \left[\begin{array}{ccc}0.5& \dots & 0.7\end{array}\right], k mod 11=9, 10\end{array}\right.$$

where $v$ is the actual speed of the spaceship and $k$ is a software counter.

We computed changes in SMR, T, and SMR/T ratio across the 4 channels for each session and participant for both the real and sham nM groups, which were used for the statistical analyses.

#### NeuroTracker

We used the so-called 3D multiple object-tracking task that requires the participant to fixate on a green dot in the middle of the screen and use peripheral vision to monitor the movements of eight yellow spheres. One of the earliest published papers on NT^51^ describes the four phases of each trial. Briefly, during the first phase of each trial, all 8 spheres appeared in yellow and were stationary. Then, the 4 target spheres that the participant must track appeared in red for 2 s, before switching back to yellow. After this, all 8 spheres started to move along a linear path through a 3D cube. The participant was asked to track the 4 target spheres over a period of 8 s. Should any sphere encounter an obstacle it bounced off that obstacle and continued along its new path. At the end of each trial, the spheres were identified with a number and the participant was asked to click on the target spheres.

### Statistical analyses

Statistical analyses were performed using SPSS Statistics Package (version 28.0, SPSS Inc., Chicago, IL, USA). All data were checked by Shapiro–Wilk’s test and visual inspection of their histograms. Log transformation was used for variables that were not normally distributed. The analyses were done on the transformed data but all variables are reported in their original, non-transformed, form as mean ± standard deviation (SD). Separate time (PRE, POST) × group (nM, NT, CON) repeated-measures analysis of variance (anovaRM) and planned post-hoc tests with Bonferroni correction for multiple comparisons were performed to assess the effects of a 12-session computer-based cognitive training program on the changes in cognitive abilities. Further anovaRM was used to evaluate the differences in EEG metrics (SMR, T, SMR/T) across the sessions. Complementary post-hoc analyses (paired-samples t-tests) were used when indicated. Cohen’s effect size, d, was also computed as appropriate. Additionally, the effect sizes of the independent variables were expressed using partial eta squared (η_p_^2^)^52^. In addition, to assess the success of the nM neurofeedback training, Mann–Whitney *U* test was performed to compare the differences between the real and sham nM groups. Furthermore, to establish the relationship between behavioral and EEG changes, a comprehensive correlation analysis was performed. Statistical significance was set at p < 0.05. Graphs were created using JASP software (version 0.17.1)^53^.

### Ethics statement

The participants received both verbal and written explanations of the experimental protocol that was in accordance with the declaration of Helsinki. After this, participants signed the informed consent document. All experimental protocols were approved by the University (Hungarian University of Sports Science, Budapest, Hungary) Ethical Committee (Approval No. TE-KEB/No36/2022).

## Results

**Figure 2:**
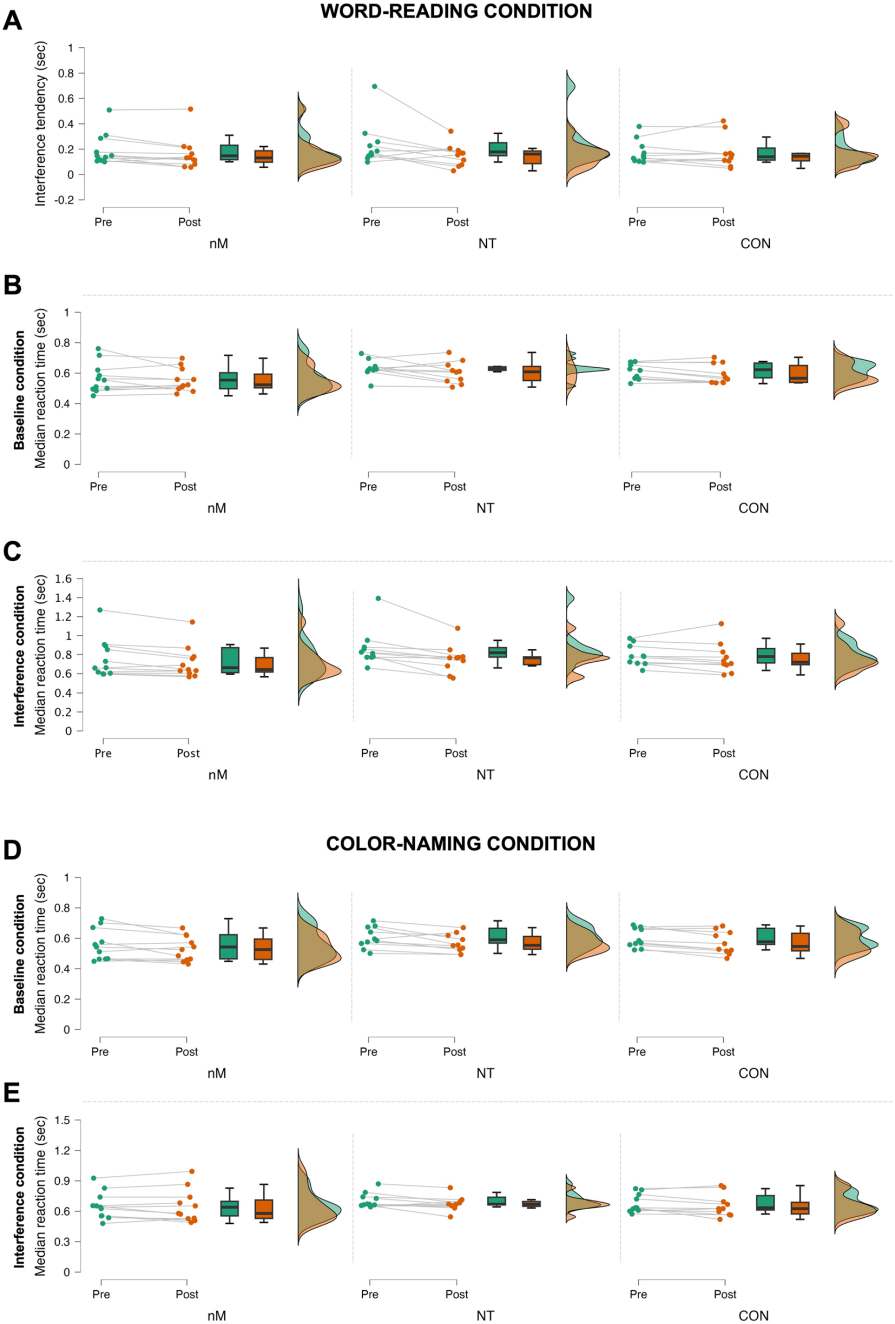
Changes in STROOP task in response to the intervention. Raincloud plots represent baseline (Pre) and post-intervention (Post) values in each group (green and orange, respectively) for interference tendency (**A**), and median reaction time of baseline and interference conditions of both STROOP word-reading (**B**, **C**, respectively) and color-naming conditions (**D**, **E**, respectively). *nM* neuroMoon, *NT* NeuroTracker, *CON* nM sham, *STROOP* Stroop test. The boxplots show the median, the upper, and lower quartiles, and the min and max value of the groups. The figure presents the cognitive measures that showed main effect of time (p < 0.05).

Raw data could be found at Supplementary Tables 1–4. Significant time main effect was found in the IF tendency (F_1,28_ = 10.943, p = 0.003, η_p_^2^ = 0.281) and median reaction time of both the BL (F_1,28_ = 6.390, p = 0.017, η_p_^2^ = 0.186) and IF task (F_1,28_ = 8.045, p = 0.008, η_p_^2^ = 0.223) of the word-reading condition of STROOP. Pairwise comparisons of pre- and post-values revealed faster interference tendency (pre: 0.204 ± 0.133 vs. post: 0.163 ± 0.113 s, Cohen’s d = 0.479) (Fig. 2A) and median reaction time for BL (0.604 ± 0.076 vs. 0.583 ± 0.073 s, d = 0.445, Fig. 2B) and IF (0.807 ± 0.178 vs. 0.746 ± 0.155 s, d = 0.761, Fig. 2C), regardless of group. In addition, time main effects with pairwise comparisons of pre- and post-values indicated faster median reaction time of both the BL (p < 0.001, d = 0.859, Fig. 2D) and IF task (p = 0.026, d = 0.420, Fig. 2E) of the STROOP color-naming condition with no between-group differences.

**Figure 3:**
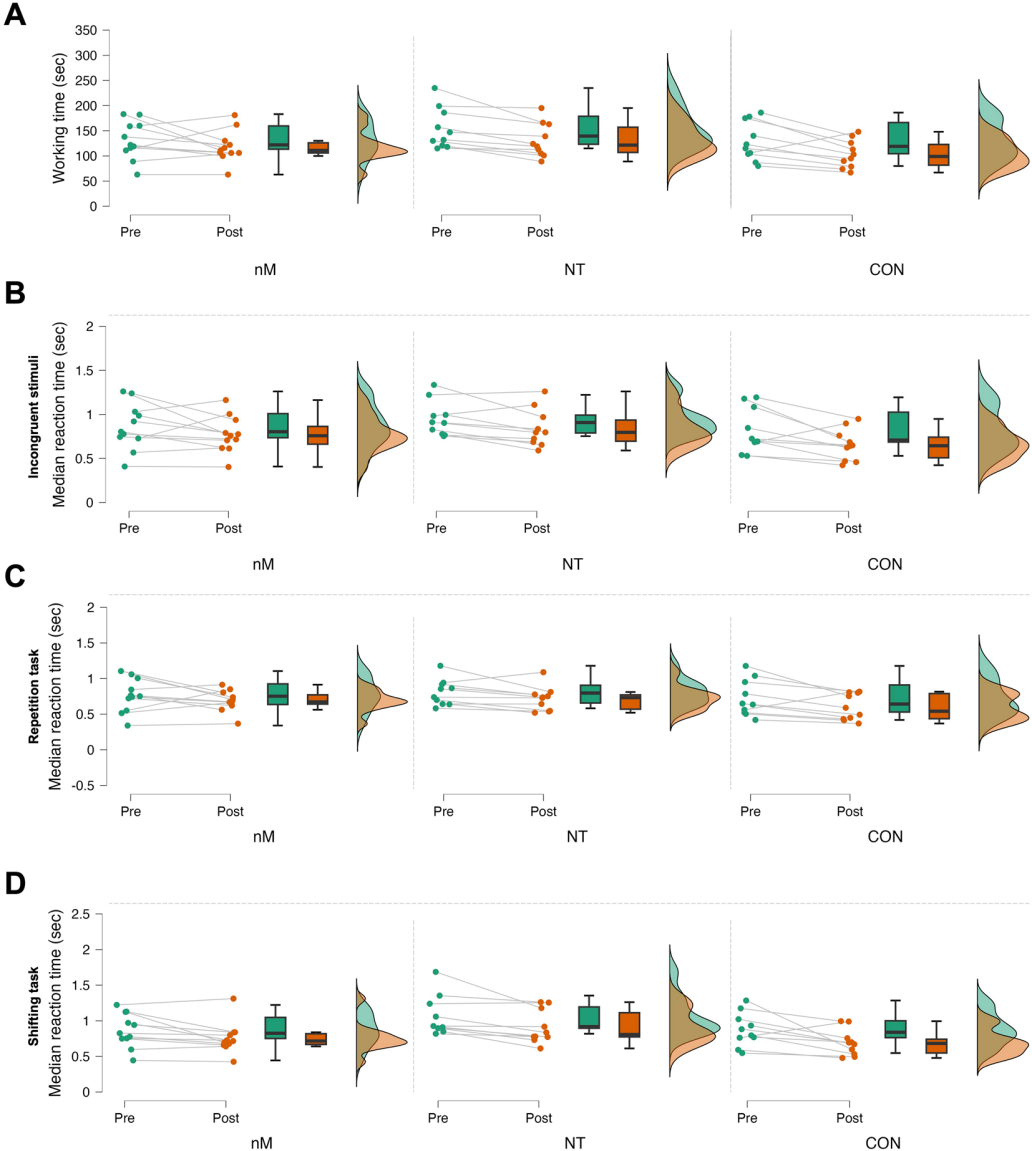
Changes in SWITCH task in response to the intervention. Raincloud plots represent baseline (Pre) and post-intervention (Post) values in each group (green and orange, respectively) for working time (**A**), and median reaction time of incongruent stimuli (**B**), repetition task (**C**), and shifting task (**D**). *nM* neuroMoon, *NT* NeuroTracker, *CON* nM sham. The boxplots show the median, the upper, and lower quartiles, and the min and max value of the groups. The figure presents the cognitive measures that showed main effect of time (p < 0.05).

Regarding SWITCH, time main effect was found in the working time (F_1,28_ = 17.968, p < 0.001, η_p_^2^ = 0.391) and the mean reaction time of the incongruent stimuli (F_1,28_ = 15.989, p < 0.001, η_p_^2^ = 0.363), repetition task (F_1,28_ = 9.573, p = 0.004, η_p_^2^ = 0.255), and shifting task (F_1,28_ = 4.558, p = 0.042, η_p_^2^ = 0.140). Pairwise comparisons of pre- and post-values revealed faster post-intervention working time (Fig. 3A) and mean reaction times for each above-mentioned variable (all p < 0.05) (Fig. 3B–D).

**Figure 4:**
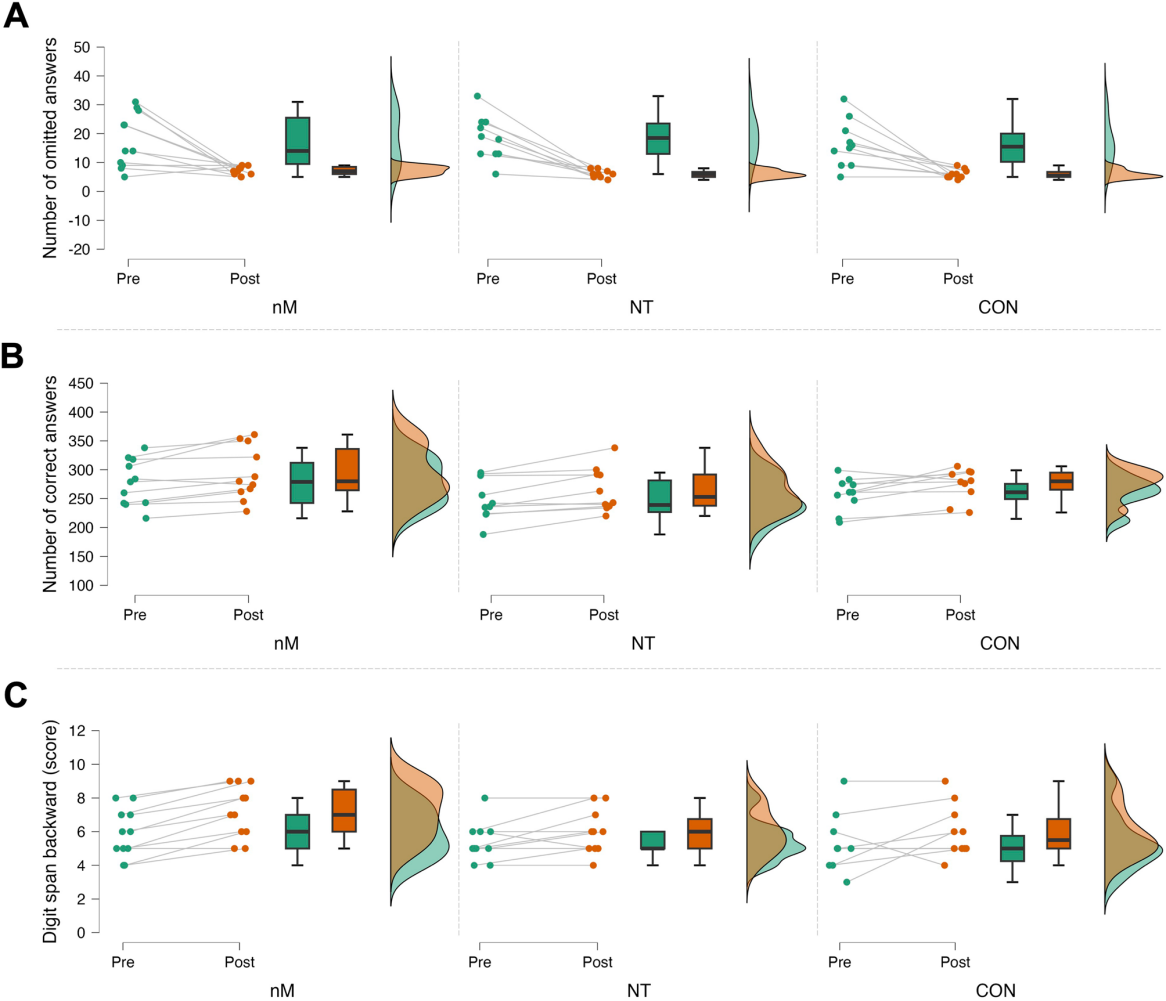
Changes in DT and DSB in response to the intervention. Raincloud plots represent baseline (Pre) and post-intervention (Post) values in each group (green and orange, respectively) for the number of omitted (**A**) and correct (**B**) answers of DT, and DSB score (**C**). *nM* neuroMoon, *NT* NeuroTracker, *CON* nM sham. The boxplots show the median, the upper, and lower quartiles, and the min and max value of the groups. The figure presents the cognitive measures that showed main effect of time (p < 0.05).

Furthermore, mixed ANOVA revealed significant time main effect for both the omitted (F_1,28_ = 71.554, p < 0.001, η_p_^2^ = 0.719) and correct (F_1,28_ = 33.541, p < 0.001, η_p_^2^ = 0.545) answers of DT with pairwise comparisons of pre- and post-values showing fewer omitted (pre: 17.5 ± 8.3, post: 6.4 ± 1.5, d = 1.311) (Fig. 4A) and more correct (pre: 261.6 ± 36.1, post: 278.6 ± 38.7, d = − 1.020) (Fig. 4B) answers post-intervention, regardless of group. All the other VTS-related cognitive test variables were non-significant (all p > 0.05).

There was a time main effect (F_1,28_ = 15.218, p < 0.001, η_p_^2^ = 0.352) in the DSB with pairwise comparisons of pre- and post-values showing larger post (6.42 ± 1.54) vs. pre (5.55 ± 1.43) scores in response to the intervention (d = − 0.801), regardless of group (Fig. 4C).

Regarding nM neurofeedback training, no differences were found between the real and sham nM groups either in EEG metrics (SMR, T, SMR/T ratio) (all p > 0.05) or game scores (p = 0.96) across the sessions (Supplementary Fig. 1).

**Figure 5:**
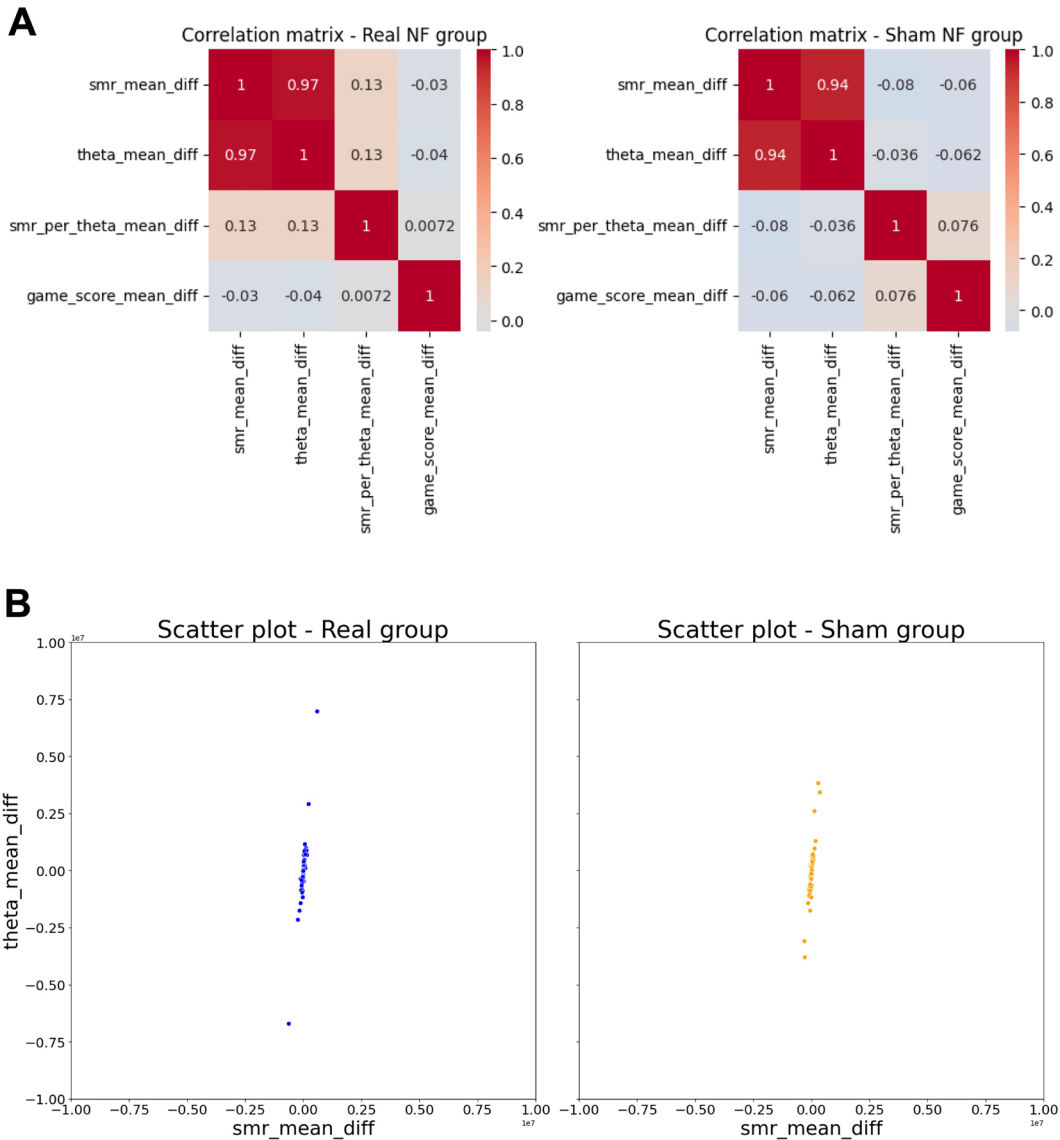
Correlation matrices for the real and sham NF groups in EEG metrics and game scores. (**A**) The correlation matrix heatmap shows the Pearson correlation coefficient (r) values for EEG variables (SMR, T, SMR/T ratio) and game scores. The positive values in red, negative in blue. It ranges from − 1 to 1, whereby − 1 means a perfect negative linear relationship between variables, 1 indicates a perfect positive linear relationship between variables and 0 indicates that there is no relationship between studied variables. (**B**) Scatterplot for the significant positive associations between SMR and T in both real and sham NF groups.

Finally, correlation analyses (Fig. 5A) revealed strong positive correlations between the differences in SMR and T across sessions in both nM (r = 0.969, p < 0.001) and sham nM (r = 0.937, p < 0.001) groups, suggesting that the increase in SMR was associated with the increase in T across sessions, regardless of group (Fig. 5B). The relationships between the changes in other EEG metrics, and EEG metrics and game score are generally weak (r < 0.3 or − 0.3) for both groups.

## Discussion

The present study aimed to investigate the feasibility and efficacy of a novel NF system, nM, on cognitive abilities from the VTS compared with one of the most popular PCT tools, NT. Given the promising results of NF in cognitive improvement, we hypothesized that nM might also be useful in athletes to enhance their cognitive performance so that the level of cognitive improvements after nM vs. NT training will not differ. In line with our hypothesis, we found only time main effects in a set of cognitive measures, including reaction time, working time, and accuracy, suggesting that PCT with nM has the potential to be used to achieve and maintain better mental performance. However, the time main effect also indicate that the sham stimulation induced similar improvements in cognitive abilities which could be due to 4 reasons: (1) the pre-test already made participants familiar enough with the cognitive tests so that they could perform the post-test with enhanced performance, (2) none of the interventions had additional learning effect, (3) even the sham stimulation induced beneficial changes in participants’ cognitive abilities, and (4) the measured variables are not the ones that could benefit the most from NF-based PCT. This is supported by the results, i.e., no differences were found between the real and sham nM groups either in EEG metrics (SMR, T, SMR/T ratio) (all p < 0.05) or game scores (p = 0.96) across the sessions; moreover, the relationships between the changes in EEG metrics and game score are generally weak for both groups. These results suggest that the NF training was not successful most probably due to the relatively short (4 weeks, 3 times a week) intervention.

The peer-reviewed literature suggests that experts are better than nonexperts in perceptual-cognitive skills^1^, which can help them to process key information at the right time to make accurate decisions during the competitions^6^. Given that neurocognitive assessment tools appear to optimize, maintain, and improve perceptual-cognitive performance, it is not surprising that their incorporation into the training regime has become widely accepted in both sports and rehabilitation. In addition, as NF seems to induce beneficial changes in cognitive functions and attention-deficit/hyperactivity disorder (ADHD)^8, 9^, engineers have begun to develop neurocognitive assessment tools using SM NF. For this aim, a Hungarian small enterprise, MindRove, has developed an EEG-based NF device, i.e., nM, with the expectation that it can be integrated into existing astronaut training to support performance outcomes. It features a flexible and adjustable headset consisting of four rigid components integrated into a headband. The 4 EEG electrodes are dry, requiring no gel or paste to be applied to the skin. Although the device is intended to be commercially available in the near future, its feasibility is not yet known. Therefore, we examined the effectiveness of nM on a comprehensive battery of VTS cognitive tests before and after a 12-session computer-based cognitive training program with either nM or NT device.

In line with our hypothesis, statistical analyses revealed only time main effects in cognitive abilities suggesting no differences between the effectiveness of nM and NT. For example, participants in both groups had faster had faster median reaction time in both the color-naming and word-reading conditions of STROOP (Fig. 2) following the training, suggesting that both NT and nM are feasible to improve cognitive flexibility and task-switching ability. In line with this, participants’ overall working time (Fig. 3A) and mean reaction time of the incongruent stimuli (Fig. 3B), repetition task (Fig. 3C), and shifting task (Fig. 3D) also improved in the SWITCH task, suggesting that these PCTs have the potential to induce beneficial changes in executive function, i.e., task-switching ability. In addition, participants in both nM and NT performed the DT with fewer omitted (pre: 17.5 ± 8.3, post: 6.4 ± 1.5, d = 1.311) (Fig. 4A) and more correct (pre: 261.6 ± 36.1, post: 278.6 ± 38.7, d = − 1.020) (Fig. 4B) answers following the PCT. Regarding the DSB cognitive test, participants in each group performed the DSB with larger post (6.42 ± 1.54) vs. pre (5.55 ± 1.43) scores following the PCT (Fig. 4C), which further supported the hypothesized beneficial effects of PCT in cognitive flexibility and task-switching ability, regardless of the method itself. Nevertheless, CON showed similar improvements in the measured cognitive variables as compared with nM and NT.

The main limitation of the present study is the lack of an inactive control group. Considering that our training programs (NT or NM) aimed to improve cognitive skills, we should have had an inactive control group to draw clear conclusions about the efficacy of the EEG-based NF device, nM, examined in the present study on improving cognitive performance. Nevertheless, because participants in the nM group showed similar improvements in cognitive abilities as compared with NT, we can suggest that nM is as feasible to optimize, maintain, and improve perceptual-cognitive performance as NT which is one of the most popular PCT tools both in sports and rehabilitation. Another limitation of the present pilot study is the relatively small sample size. Future studies will need to recruit more participants to increase statistical power, as some of the significant changes in response to PCT training in the present study may be due to the changes in inter-subject variability. The lack of (1) differences between the real and sham nM groups and (2) relationships between the changes in EEG metrics and game score could be most probably due to the relatively short intervention, therefore, future studies should clarify whether performing NF training for a longer period could improve perceptual-cognitive performance.

Moreover, a few additional questions are raised. The efficacy of NF depends both on the frequencies to be enhanced and/or suppressed and on the location of the specific rhythms^54, 55^. For example, in archery, the increase in performance may be associated with activation of the right hemisphere and selective inhibition of left temporal lobe activity, or in the supplementary motor field, alpha suppression may facilitate the automation of movements such as golf putting^56^ or de-automatized walking^57^. As far as the frequency bands are concerned, their usefulness depends on the task in question. The wrong type of activity may not only be ineffective but may also be harmful, i.e., it may either work against a specific performance measure that is not ‘similar enough’ to the competence to be developed (therefore, it is crucial to choose performance metrics of appropriate quality/type) or the competence itself^54^. One of the main features of our research was the relatively high diversity in terms of sports, as participants played both individual and team sports, and their background may have an impact on their learning curve and their performance in pre- and post-tests. Another interesting concern is the extent to which the results of the NF program are affected by the irregularity of the frequency of sessions, as the length of the gaps between sessions has an impact on the effectiveness of NF programs of the same length (i.e., with the same number of sessions)^58^. Despite our best efforts to keep the frequency of sessions constant, this was not always possible due to the time constraints of our subjects and some sessions had to be postponed due to illness.

Furthermore, given that nM was developed with the expectation that it can be integrated into existing astronaut training to support performance outcomes, future studies should determine changes in real astronaut-related performance in response to nM training preferably while acquiring biosignals.

## Conclusions

In conclusion, a series of cognitive measures showed similar improvements following PCT with nM as compared with NT suggesting that this system may support the achievement and maintenance of improved mental performance in complex environments under challenging conditions. Future studies should determine its feasibility in real-world performance outcomes for astronauts to clearly identify its validity.

### Supplementary Information

Supplementary Information.

## Data Availability

The datasets used and/or analyzed during the current study are presented within the manuscript and/or additional supporting files and are also available from the corresponding author on reasonable request.

## Abbreviations

CON: Sham neuroMoon group
DSB: Digit span backwards test
DT: Determination test
EEG: Electroencephalography
NF: Neurofeedback
nM: NeuroMoon
NT: NeuroTracker
PCT: Perceptual-cognitive training
anovaRM: Repeated-measures analysis of variance
RT: Reaction test
SM: Sensorimotor
SMR: Sensorimotor rhythm
SMR/T: Sensorimotor rhythm-theta ratio
STROOP: Stroop test
SWITCH: Task-switching test
T: Theta
TBR: Theta-beta ratio
TMT: Trail-making test
VTS: Vienna test system
