# Supplementary material for: Feasibility of a novel neurofeedback system: a parallel randomized single-blinded pilot study
Source: Sci Rep. 2023 Oct 13;13:17353. doi: 10.1038/s41598-023-44545-1 (PMC10576027; doi:10.1038/s41598-023-44545-1)
Supplement: Supplementary file 1 — Supplementary Information. [file 41598_2023_44545_MOESM1_ESM.docx]

*Supplementary material*

**Title:** Feasibility of a novel neurofeedback system - A parallel randomized single-blinded pilot study

**Journal name:** nature Scientific Reports

**Authors:** Dávid Horváth^1, 2, *^, János Négyesi^1, 2, 3^, Melinda Rácz^4, 5, 6, 7^, Tamás Győri^2, 8^, Zsolt Matics^2^, Artyom Puskin^5^, János Csipor^5^, Levente Rácz^1^

**Affiliations:** ^1^Department of Kinesiology, Hungarian University of Sports Science, Budapest, Hungary; ^2^Fit4Race Kft., Budapest, Hungary; ^3^Department of Medicine and Science in Sports and Exercise, Tohoku University Graduate School of Medicine, Sendai, Japan; ^4^Research Centre for Natural Sciences, Eötvös Loránd Research Network, Budapest, Hungary; ^5^MindRove Kft., Győr, Hungary; ^6^János Szentágothai Doctoral School of Neurosciences, Semmelweis University, Budapest, Hungary; ^7^Selye János Doctoral College for Advanced Studies, Semmelweis University, Budapest, Hungary; ^8^Department of Psychology and Sport Psychology, Hungarian University of Sports Science, Budapest, Hungary

**Corresponding author:**

Dávid Horváth

e-mail: [**david.horvath@fit4race.com**](mailto:david.horvath@fit4race.com)

**Supplementary Figure 1:** EEG metrics across the sessions

**
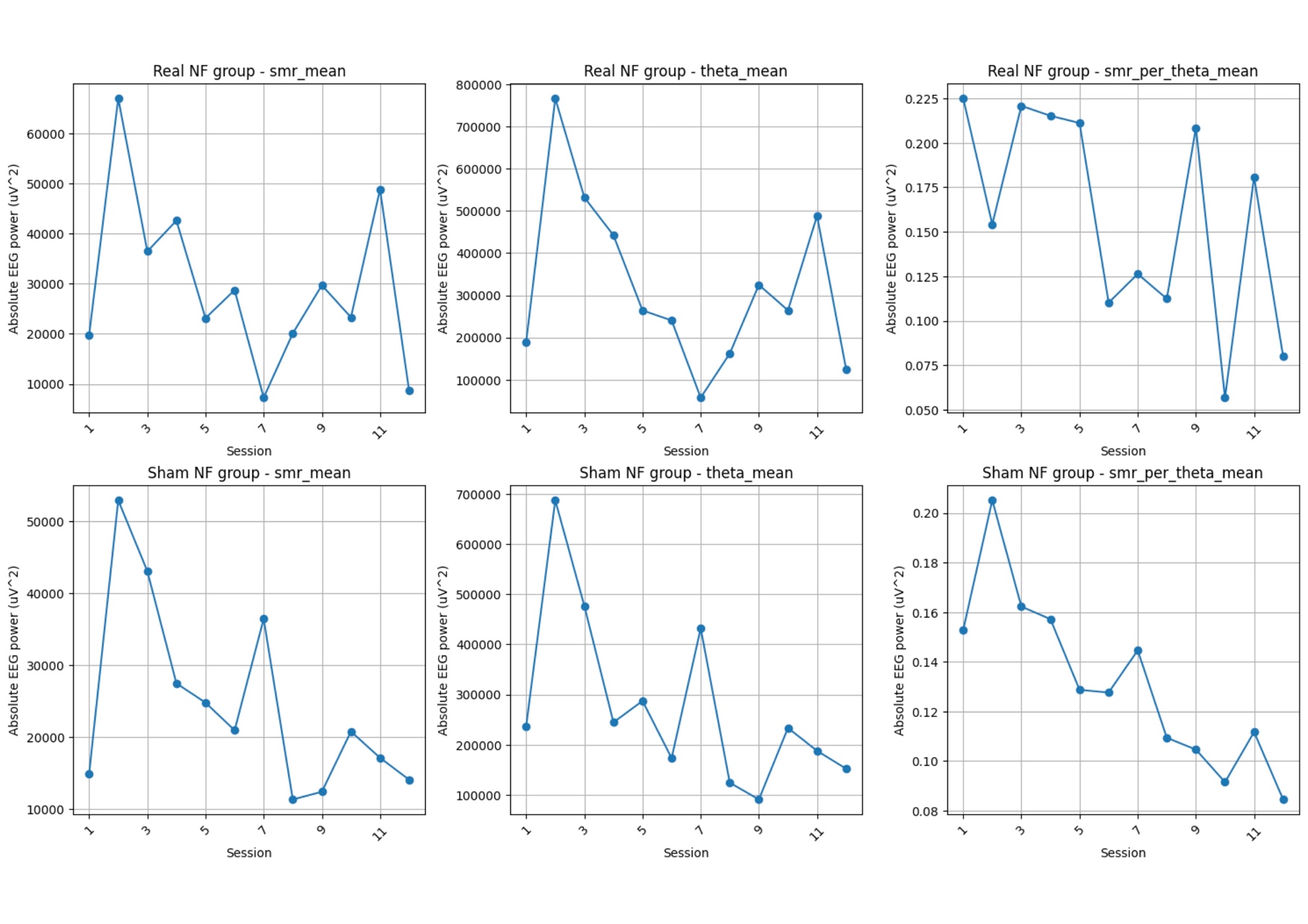
**

**Supplementary Table 1:** Supplementary dataset for DSB, TMT, and DT values

**Supplementary Table 2:** Supplementary dataset for RT, and STROOP Reading values

**Supplementary Table 3:** Supplementary dataset for STROOP Naming values

**Supplementary Table 4:** Supplementary dataset for SWITCH values
